# Errors in Methods and Results

**DOI:** 10.1001/jamanetworkopen.2026.41171

**Published:** 2026-09-29

In the Original Investigation titled, “Neurodegenerative Disease Death Certification Among National Football League Players With Dementia,”^1^ published September 1, 2026, there was an error in the recruitment dates in the Methods. The initial date of recruitment has been changed from February 15, 2028, to February 15, 2008. Additionally, in the Results the odds ratio (OR) for neurodegenerative disease death certification in memory care or nursing homes was changed from “(OR, 2.30; 95% CI, 1.16-4.56)” to “(OR, 2.29; 95% CI, 1.16-4.56).” This article has been corrected.^1^
